# Health–Economic Impact Attributable to Occurrence of Pleurisy and Pneumonia Lesions in Finishing Pigs

**DOI:** 10.3390/vetsci11120668

**Published:** 2024-12-20

**Authors:** Clarisse S. Malcher, Fernando A. M. Petri, Laiza P. Arruda, Gabriel A. de Aguiar, Gabriel Y. Storino, Karina Sonalio, Leonardo T. Toledo, Flávio Hirose, Luís Guilherme de Oliveira

**Affiliations:** 1 School of Agricultural and Veterinary Sciences, São Paulo State University (UNESP), Jaboticabal 14884-900, SP, Brazil; cs.malcher@unesp.br (C.S.M.); fernando.petri@unesp.br (F.A.M.P.); laiza.arruda@unesp.br (L.P.A.); ga.aguiar@unesp.br (G.A.d.A.); gabriel.storino@unesp.br (G.Y.S.); karina.sonalio@unesp.br (K.S.); 2 Faculty of Veterinary Medicine, Ghent University, 9820 Merelbeke, Belgium; 3 Department of Veterinary Medicine, Federal University of Viçosa, Viçosa 36570-900, MG, Brazil; leonardo.teofilo@ufv.br; 4 Ourofino Animal Health, Cravinhos 14140-000, SP, Brazil; flavio.hirose@ourofino.com

**Keywords:** *Actinobacillus pleuropneumoniae*, ADG, intensive pig farming, *Mycoplasma hyopneumoniae*, *Pasteurella multocida*, PRDC, SPES

## Abstract

Respiratory diseases, such as pleurisy and pneumonia, cause significant health and economic losses in pig production. This study evaluated 867 finishing pigs from a farm with a history of respiratory issues, using macroscopic lesion scoring (SPES and CVPC), histopathological analysis, qPCR diagnostics, and economic modeling. Severe pleurisy (scores 3 and 4) was observed in 42.1% of carcasses and was strongly correlated with higher bacterial loads of *Mycoplasma hyopneumoniae*, *Actinobacillus pleuropneumoniae*, and *Pasteurella multocida*. Severe lesions reduced the average daily gain (ADG) and carcass weight, leading to increased production costs and lower profitability. Economic analysis revealed that pigs with higher pleurisy scores incurred an additional cost per kg (USD 1.29 vs. USD 1.32 for milder cases), reduced total revenue by 1.36%, and decreased return on investment (ROI) from 5.33% to 3.90%. These findings emphasize the critical impact of respiratory diseases on profitability and the necessity of robust health management strategies, including vaccination and enhanced biosecurity, to minimize economic losses in swine farming.

## Simple Summary

This study evaluated the health and economic impacts of respiratory diseases, specifically pleurisy and pneumonia, in finishing pigs. These conditions are common in intensive pig farming and lead to reduced growth rates, lower carcass quality, and significant economic losses. In this research, pigs from a farm with a history of respiratory diseases were monitored through their finishing phase, with lung and pleura samples collected at slaughter. The results showed a high prevalence of lung lesions, notably bronchopneumonia, with frequent co-infections by pathogens such as *Mycoplasma (M.) hyopneumoniae* and *Actinobacillus (A.) pleuropneumoniae*. The severity of pleurisy lesions correlated with lower average daily gain (ADG) and higher production costs. Economic analysis indicated that pigs with severe pleurisy yielded lower profits due to reduced carcass weight and increased costs, emphasizing the need for effective disease management. This study highlights the significant toll that respiratory diseases take on pig production, both in animal health and profitability, underscoring the importance of preventive measures to improve welfare and economic outcomes.

## 1. Introduction

Porcine respiratory disease complex (PRDC) is a significant challenge for the global pig industry, especially as it affects growing and finishing pigs. This multifaceted condition arises from a combination of pathogens and environmental stressors affecting the respiratory system of pigs [1]. This clinical condition imposes considerable economic impacts on the swine industry. Infection by *M. hyopneumoniae* typically results in several production impacts, including decreased average daily gain (ADG), increased mortality rate, reduced feed efficiency, and increased need for antibiotics to control the disease, as well as losses in carcass quality or even condemnation due to lung lesions [2]. The most frequently observed lung lesions in slaughterhouses result from pneumonia and pleurisy [3] and may be attributed to significant losses around the world [4,5,6].

Cranioventral pulmonary consolidation (CVPC) and pleurisy lesions in slaughter pigs are typical of infections by *M. hyopneumoniae* and *Actinobacillus pleuropneumoniae* (*A.*) *pleuropneumoniae*, respectively [1]. These are significant lesions, considering other pathogens that may cause similar lesions and/or also contribute to these lesions. As a result, infected animals become more susceptible to infections by other respiratory diseases [3]. In herds with high serological prevalence rates of *A. pleuropneumoniae*, finished pigs presented more severe lung and pleuritic lesions at slaughter, affecting up to 90% of the animals [7]. Respiratory diseases in pigs, particularly cranioventral pulmonary consolidation (CVPC) and pleurisy, significantly impair production efficiency by reducing growth rates, carcass weight, and meatiness while increasing costs associated with prolonged fattening periods and reduced feed conversion. Severe lung lesions (≥15.1%) can lower average daily carcass weight gain by up to 4.6% and result in economic losses of up to EUR 11.53 per pig under average market conditions [8].

The slaughterhouse plays a crucial role in complementing the clinical history of pigs affected by the porcine respiratory disease complex (PRDC), enabling sample collection for diagnosing key diseases [9,10]. According to Przyborowska-Zhalniarovich et al. (2023) [11], the effectiveness of PRDC control measures can be evaluated by analyzing the prevalence and severity of respiratory diseases. Diagnosing pleurisy in pigs is challenging due to the often absence of clinical signs, with lesions typically identified only at slaughter [1].

Pleurisy associated with lung nodules is primarily caused by *A. pleuropneumoniae* [12,13], transmitted by asymptomatic carriers through direct contact or aerosols over short distances [14]. Primary pleurisy is linked to pathogens such as *M. hyopneumoniae*, *A. pleuropneumoniae*, and swine influenza A virus (IAV) [1]. In a study by De Conti et al. [15], *Pasteurella* (*P.*) *multocida* type A was isolated in 54.2% of lung lesions, with 68% of samples showing histopathological lesions suggestive of multiple pathogens, including *M. hyopneumoniae* and IAV.

Primary pathogens predispose to secondary infections caused by *P. multocida*, *Glasserella* (*G.*) *parasuis*, and *Streptococcus* (*S.*) *suis* [16]. The most common form of pleurisy at slaughterhouses is fibrinous pleurisy, a chronic inflammatory process where protein-rich exudative fluid accumulates in the pleural space [17]. Nascimento et al. (2018) [18], evaluated 4536 pigs from multiple Brazilian regions during 2013 and 2014 and found pleurisy lesions in 10.6% of animals (482 pigs). Among the causative agents, *G. parasuis* was the most prevalent (55%), followed by *P. multocida* (31%), *A. pleuropneumoniae* (7%), *S. suis* (6%), and *Bordetella (B.) bronchiseptica* (1%). *G. parasuis* was primarily associated with the nursery phase (82.57%), while *P. multocida* (83.93%) and *A. pleuropneumoniae* (93.52%) were predominant in the growth phase. In contrast, the prevalence of *S. suis* and *B. bronchiseptica* was independent of the pigs’ growth phases.

Mores et al. (2017) [19] analyzed 3,521,824 pig carcasses from a slaughterhouse in Southern Brazil (2010–2014) and found 7.3% (258,062) diverted to the Final Inspection Department (DIF) due to pleuritis/pericarditis. Of these, 94.8% were approved for domestic sales, 3.72% for preservation, and 1.45% for rendering. Similarly, Coldebella et al. (2018) [20] reported pleural adhesion as the leading cause of carcass condemnation in Brazil, affecting 3.72% of slaughtered pigs. In practice, the DIF (Department of Inspection of Animal Products) operates through activities of inspection, monitoring, and control of establishments and animal-based products. In slaughterhouses registered with the Federal Inspection System (SIF), product samples are collected and sent to laboratories for microbiological, physicochemical, and residue analyses to ensure foods are safe for consumption.

Based on the context presented, this study evaluates the productive and financial impact of pneumonia and pleurisy lesions in pigs, highlighting the role of respiratory diseases during the finishing phase and their economic consequences for pig farmers.

## 2. Materials and Methods

### 2.1. Ethics Committee

All the procedures described in this study using pigs were submitted and approved by the Animal Use Ethics Committee (CEUA) of the School of Agricultural and Veterinary Sciences of São Paulo State University (Unesp—Campus Jaboticabal), under protocol nº #22/003581.

### 2.2. Animals, Epidemiological Unit, and Study Design

A commercial pig farm with a history of respiratory diseases, located in the State of São Paulo, Brazil, was selected for study. The experimental herd consisted of 1050 pigs housed in a shed containing 8 stalls during the finishing phase. Each stall was divided into 8 collective pens, with approximately 130 pigs per pen, ensuring a stocking density of 1.2 m^2^ per pig. The floor was fully slatted concrete, designed for waste management, and the shed was equipped with automatic feeders and drinkers to provide free access to age-appropriate water and feed. Ventilation was natural, supported by side curtains. At 63 days of age (Day 0—D0), pigs were individually identified and kept in collective pens.

The farm’s vaccination protocol included vaccines against Porcine Circovirus Type 2 (PCV-2) and *M. hyopneumoniae* (CIRCUMVENT^®^ PCV M, MSD Animal Health, Rahway, NJ, USA) administered at 21 and 42 days of age (2 mL, via intramuscular in the ear base), along with an autogenous vaccine for *A. pleuropneumoniae*. Other medications in this lot included florfenicol 40% (Maxflor^®^, Virbac, São Paulo, Brazil) given to 57% of pigs (599/1050) via intramuscular (IM) injection at a 20 mg/kg dose each 48 h, and ceftiofur (Minoxel^®^ Plus, Lapisa, São Paulo, Brazil) at 1–3 mg/kg for three days. Tiamulin hydrogen fumarate 45% was administered via water to all pigs in the barn. Also, pigs were managed according to the farm’s standard routine. The study observed farm-standard management practices under natural conditions to ensure practical relevance.

### 2.3. Blood Sampling and Body Weight Measurement

Blood samples and body weights were collected at the start of the finishing phase (63 days of age, D0) and prior to slaughter (153 days, D90) according to the slaughterhouse routine. Blood samples were taken by puncturing the jugular vein using 25 × 0.8 mm needles and vacuum tubes (BD Vacutainer^®^, Franklin Lakes, NJ, USA) after antisepsis with 70% alcohol. Samples were centrifuged at 1500× *g* for 10 min (Centrifuge 5804 R, Eppendorf^®^, Hamburger, Germany), and serum aliquots were stored in 2 mL sterile microtubes (Kasvi, Pinhais, Brazil) at −20 °C until further processing.

Body weight data were used to calculate the average daily gain (ADG) for the batch. The ADG was determined by subtracting the initial weight (IW) from the final weight (FW) and dividing the value obtained by the number of days in the phase (ADG = (FW − IW)/days) [21].

### 2.4. Slaughtering and Scoring of Pneumonia and Pleuritis

At the end of the finishing phase, all the pigs were slaughtered at a slaughterhouse registered with the Federal Inspection System (SIF) located in the State of São Paulo, with a daily slaughter flow of 1200 pigs, according to the methods established by ordinance No. 711, 01 November 1995, of the Ministry of Agriculture, Livestock, and Food Supply [22]. The procedures were established conveniently according to the routine of the slaughterhouse flow.

After evisceration, lungs and carcasses were identified and classified according to the Slaughterhouse Pleurisy Evaluation System (SPES) described by Dottori et al. (2007) [23]. This method categorizes pleurisy lesions into five scores (0–4) (Table S1). We classified the collected samples according to this score, considering the extent and location of pleural adherence to the cavity chest, as employed by other studies [9,10]. Cranioventral pulmonary consolidation (CVPC) lesions were scored using the method of Piffer and Britto (1991) [24], with each of the seven lung lobes graded from 0 to 4 (Table S2). The average lesion score was calculated as the total lobe scores divided by the number of evaluated lungs.

Additionally, to assess *A. pleuropneumoniae*-associated pleuritis, the *A. pleuropneumoniae*-Associated Pleuritis Index (APPI) was calculated. The APPI considers the frequency of pleuritis lesions with scores above 2 in a batch, multiplied by the average pleuritis lesion score in animals with an SPES score of above 2, providing insight into lesion prevalence and severity within the batch.

In the SIF, after evisceration, the carcasses presenting severe lesions are diverted to the Final Inspection Department (DIF), where they are subjected to a secondary line of slaughter, with a federal veterinary inspector checking and assessing the macroscopic lesions, and then they allow or condemn the carcass. In our study, we were able to collect the biological samples of the pleuritic carcasses at this point, as we describe in Section 2.5.

### 2.5. Collection of Tissue Samples at Slaughter and Histopathological Analysis

After the macroscopical evaluation of the carcasses directed to the DIF, pleural and lung tissues were collected, including bronchi and parenchyma from the observed lesions. Smaller portions were placed in 2 mL sterile DNAse and RNAse-free microtubes (Eppendorf, Hamburger, Germany) for qPCR, while larger fragments (~1 cm^3^) were fixed in 10% buffered formalin for histopathological analysis. Samples were labeled and preserved in liquid nitrogen during transport to the Swine Medicine Laboratory at FCAV—Unesp Jaboticabal, where they were kept frozen at −80 °C until further analyses. Tissue collection was performed using sterilized tools to maintain sample integrity for molecular and pathological examinations.

Fragments for histopathological analysis were fixed in 10% formalin for 24 h, transferred to 70% alcohol, dehydrated, clarified, embedded in paraffin, sectioned (2–5 µm), and stained with hematoxylin and eosin (HE). Alveolar exudates were classified as suppurative (neutrophil predominance), non-suppurative (mononuclear inflammatory cell-dominant), or mixed (intermediate) based on Bochsler and Slauson (2002) [25]. Acute lesions featured neutrophil dominance, extensive edema or fibrin exudation, and the absence of chronic signs. Chronic lesions were characterized by fibroplasia, BALT (bronchial-associated lymphoid tissue) hyperplasia (+ to ++++), bronchial/bronchiolar epithelial hyperplasia, and lymphocyte- and plasma-cell-dominant infiltrates [26].

### 2.6. DNA Extraction and qPCR Endogenous Gapdh Gene

DNA was extracted using 30 mg of tissue following the Tris-HCl protocol by Kuramae-Izioka et al. (1997) [27]. DNA concentration and purity were measured using a NanoDrop^®^ One Spectrophotometer (Thermo Fisher Scientific^®^, Wilmington, NC, USA). Samples with a 260/280 purity ratio outside 1.8 to 2.0 underwent a second extraction [28]. To ensure quality and avoid the presence of inhibitors or false negatives in qPCR, the extracted DNA was tested using the *gapdh*-F (5′-TCCTGGGCTACACTGAGGAC-3′) and gapdh-R (5′-ACCAGGAAATGAGCTTGACG′) primers, targeting a 123 base-pair fragment of the mammalian *gapdh* gene (Almeida et al. 2020) [29]. DNA samples were stored at −20 °C until qPCR analysis.

### 2.7. Detection of A. pleuropneumoniae, M. hyopneumoniae, and P. multocida by qPCR Multiplex

For pathogen detection, qPCR targeted the *omlA* gene of *A. pleuropneumoniae* (F: 5′-CCTGTCCGTGTCCTTTGC-3′ and R: 5′-GGGGACGTAACTCGGTGA-3′; probe: 5′-Cy5-CGATGAACCCGATGAGCCGCC-3′-IB^®^RQ) [30]; the *p102* gene of *M. hyopneumoniae* (F: 5′-AAGGGTCAAAGTCAAAGTC-3′ and R: 5′-AAATTAAAAGCTGTTCAAATGC-3′; probe: 5′-FAM-AACCAGTTTCCACTTCATCGCC-§BHQ2-3) [31]; and the *kmt1* gene of *P. multocida* (F: 5′-GGGCTTGTCGGTAGTCTTT-3′ and R: 5′-CGGCAAATAACAATAAGCTGAGTA-3′-IB^®^RQ) [32].

The qPCR reaction included 2 μL of DNA sample, 0.3 μM hydrolysis probe, 0.5 μM primers, 1x GoTaq^®^ Master Mix (Promega^®^, Madison, WI, USA), and sterile ultrapure water (nuclease-free water, Promega^®^, USA) in a 10 μL volume. Amplifications were performed on a CFX96™ Real-Time PCR Detection System (Bio-Rad, Marnes-la-Coquette, France) with an initial denaturation at 95 °C for 3 min, followed by 39 cycles of 95 °C for 15 s and 57.2 °C for 30 s, as adapted by Petri et al. (2023) [6].

Each sample was tested in duplicate, and the results were valid only if the standard deviation of quantification cycles (Cq) was ≤0.5; samples exceeding this were retested in triplicate. Fluorophores FAM (*p102*), Cy5 (*omlA*), and TxR (*kmt1*) were analyzed using Bio-Rad CFX Manager software (v 3.0, Bio-Rad, France). Serial dilutions of gBlock^®^ containing the target sequence (1 × 10^7^ to 1.0 × 10^1^ copies/μL) provided a standard curve for DNA quantification and served as positive controls. Data included average quantification values, Cq, and initial sample quantification (SQ). All qPCR assays followed MIQE (Minimum Information for Publication of Quantitative Real-Time PCR Experiments) guidelines [28].

### 2.8. Detection of IgG Anti-M. hyopneumoniae Antibodies

Specific antibodies IgG anti–*M. hyopneumoniae* were measured in two points: at housing in the finishing phase and before slaughter. Detection was performed with a 2-well indirect ELISA (Civtest Suis Mhyo; HIPRA), with high specificity and sensitivity, following the manufacturer’s protocol.

Plate validation followed manufacturer criteria: mean optical density(OD) of the positive control (CTR+) was assessed to be ≥0.6; the difference between the mean OD of the positive control and the mean OD of the negative control (CTR+ minus CTR–) was assessed as ≥ 0.5; and the difference between the OD of the positive diluent control and the OD of the negative diluent control was evaluated. The index reactivity with positive control (IRPC) was calculated for each sample; IRPC < 35 indicated a negative, and IRPC >35 was positive.

### 2.9. Economic Analysis

An estimated economic analysis assessed the impact of severity pleurisy in finishing pigs on profitability using the method of Alves et al. (2022) [33]. Variables included total production cost, cost per kilogram, total revenue (TR), economic profit (EP), benefit–cost ratio (BCR), and return on investment (ROI). Using the Ceteris Paribus principle, production costs were assumed constant across the batch, isolating the effect of pleurisy on profitability indicators [34]. Calculations considered both live pig sales and adjusted carcass weight (estimated yield: 80%) for comparison. Market prices from the São Paulo Association of Pig Farmers (APCS) for October 2023 were used.

### 2.10. Statistical Analysis

The Shapiro–Wilk and Bartlett tests were used, respectively, to assess data normality. For the Pneumonia Index (PI), the average percentages of pleurisy and pulmonary consolidation were determined using analysis of variance (ANOVA), followed by Tukey’s test (*p* < 0.05), to identify significant the differences between the means of variables in different pleurisy groups. To meet the normality criteria, the non-parametric Kruskal–Wallis test was used, followed by Dunn’s test. Correlations between pairs of variables were analyzed using Pearson’s correlation coefficient. Statistical tests were performed using R software version 3.6.0 (R Core Team, Vienna, Austria, 2023), and graphs were plotted using GraphPad Prism 10.2.2 software (La Jolla, CA, USA).

Additionally, a linear mixed-effect model was structured to analyze the random effects of the farm (pens) while focusing on the fixed effects of score pleurisies and gender of animals on the average daily gain (ADG) in pigs. The model was specified as follows:

*ADG_ij_* = *β*_0_ + *β*_1_(*Gender_i_*) + *β*_2_(*Score_i_*) + *u_j_* + *ϵ_ij_*

where

- *ADG_ij_*: the response variable, corresponding to the average daily gain;
- *Gender_i_*: the fixed effect of sex, categorized as female (reference) or male;
- *Score_i_*: the fixed effect of lesion scores (continuous variable);
- *u_j_*: the random intercept for pen *j*, assuming *u_j_*∼*N*(0, *σ*^2^_*Pen*_);
- *ϵ*_*ij*_: the residual error term, assuming *ϵ_ij_*∼*N*(0, *σ*^2^_*Residual*_).

The model was fitted using the restricted maximum likelihood (REML) method via the *lme4* package in R (R Core Team, 2023).

## 3. Results

### 3.1. Animals Included in the Study

Initially, 1050 pigs were weighed individually during the early phase of the experiment conducted on the farm. However, the final sample for analysis was adjusted due to interference from the slaughterhouse handling, as presented in Table 1. Of the 1050 pigs, 151 lost their identification tags (ear tags), and 26 pigs died on the farm from causes unrelated to pleurisy. The remaining 867 pigs proceeded to the slaughter line, where it was possible to evaluate and classify the lungs and pleura macroscopically.

### 3.2. Body Weight and Average Daily Gain

The body weight and carcass weight of a total of 163 pigs were evaluated across the sampling days, regarding the pleurisy score (SPES), divided into four groups. Additionally, we found that the initial body weight of pigs with a score of 1 had a higher initial body weight (32.11 ± 6.37 kg) compared to groups 3 and 4 (*p* = 0.007). No significant differences were observed in the final body weight (*p* = 0.634), ADG (*p* = 0.998), carcass weight (*p* = 0.07), or meatiness percentage (*p* = 0.13) across pleurisy scores (Table 2).

When analyzing the impact of the ADG considering the influence of disease severity according to the sex of animals, we found differences between males and females across the scores of pleurisies.

### 3.3. Gross Lesion Classification of Pneumonia and Pleuritis

Out of the 867 carcasses evaluated from the pigs initially identified on the farm, 3.58% (31/867) were absent of pleurisy, while 20.99% (182/867) and 33% (286/867) presented pleurisy scores of 1 and score 2, respectively. The most severe presentation of the condition was observed in 28.7% (249/867) and 13.4% (116/867) of pigs with SPES scores of 3 and 4, respectively. Regarding the other categories of lesions, 62.28% of the lungs (540/867) presented cranioventral pulmonary consolidation (CVPC) lesions, 24.6% (213/867) presented abscesses lesions, and 13.6% (118/867) presented App-like lesions in any lobe of the evaluated lungs.

### 3.4. Microscopic Lung Lesion Evaluation

A total of 205 lung samples were analyzed for histopathological lesions, revealing a variety of bronchopneumonia types and BALT hyperplasia classifications. Acute mixed bronchopneumonia (AMBP) was the most common lesion, affecting 44.9% of the samples (92/205). Non-suppurative acute bronchopneumonia (NSABP) was also frequent, observed in 33.7% of the samples (69/205), and chronic bronchopneumonia (CRBP) and epithelial hyperplasia (EPH) each occurred in 8.29% of the samples (17/205). The less frequent lesions included acute suppurative bronchopneumonia (ASBP) in 5.37% (11/205), subacute bronchopneumonia (SUBBP) in 2.44% (5/205), and necrotizing pneumonia (NP) in 0.97% (2/205). The BALT (bronchial-associated lymphoid tissue) hyperplasia was present in 98.5% of samples, with classifications ranging from mild (+) to extensive (++++). Mild (+) and moderate (++) hyperplasia were most prevalent, observed in 30.5% (62/203) and 45.8% (93/203) of the samples, respectively. Finally, marked (+++) hyperplasia was seen in 20.2% (41/203), while extensive (++++) hyperplasia occurred in 2.9% (6/203). The results are presented in Table 3.

### 3.5. Detection of A. pleuropneumoniae, M. hyopneumoniae, and P. multocida

Regarding the bacterial loads in the lung and pleural samples, *M. hyopneumoniae* presented higher bacterial loads (copies/µL) in samples with SPES scores of 3 and 4, with those with a score of 3 showing significantly higher loads (7.0 × 10^2^ ± 1.94 × 10^3^) than those with scores of 1 and 2. No significant differences were observed in pleural samples among the groups, although those with an SPES score of 2 had the highest mean (2.66 × 10^2^ ± 7.64 × 10^2^). Additionally, the *A. pleuropneumoniae* in lung samples with score 3 had the highest bacterial load (3.23 × 10^3^ ± 2.84 × 10^4^), significantly differing from groups with lower pleurisy scores. In pleural samples, bacterial loads did not significantly differ, although samples with an SPES score of 3 had a higher load (2.90 × 10^3^ ± 1.79 × 10^4^). Finally, the *P. multocida* in lung samples with score 3 exhibited a markedly higher load (2.30 × 10^4^ ± 1.76 × 10^5^), with significant differences compared to other groups, and no significant differences were found between groups in pleural samples, but samples with an SPES score of 2 had the highest pleural load (2.32 × 10^2^ ± 1.29 × 10^3^). The results with respective significant differences and representative graphs are presented in Table 4 and Figure S1.

**Figure 1:**
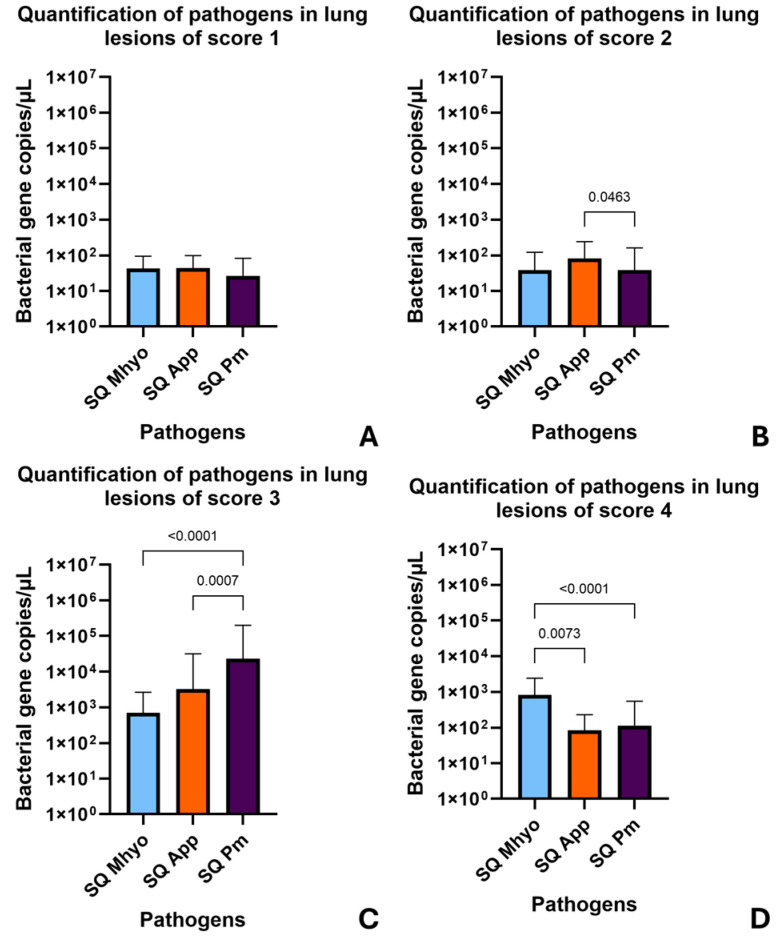
Variation in the bacterial prevalence in lung samples in each pleurisy score (SPES) group. Bar graphs show the estimated quantification of each pathogen in samples with an SPES score of (**A**) 1, (**B**) 2, (**C**) 3, and (**D**) 4. Differences were observed among *Mycoplasma hyopneumoniae* (Mhyo), *Actinobacillus pleuropneumoniae* (App), and *Pasteurella multocida* (Pm) in starting quantification (SQ) values. The Kruskal–Wallis test was used (*p* < 0.05).

Regarding the prevalence of each bacterial load according to the pleurisy score, *A. pleuropneumoniae* showed the highest bacterial load in the lung samples (5.37 × 10^1^ ± 5.22 × 10^1^), making it the most prevalent pathogen in samples with a score of 1. In samples scoring 2, *A. pleuropneumoniae* also had the highest mean load in the lungs (8.07 × 10^1^ ± 1.60 × 10^2^), presenting significant differences with loads of *P. multocida*. On the other hand, *P. multocida* was markedly more prevalent in the lung samples with SPES score 3, exhibiting the highest bacterial load among all groups (2.30 × 10^4^ ± 1.76 × 10^5^), significantly higher than other pathogens. Finally, *M. hyopneumoniae* was the most prevalent pathogen in the lungs in samples with a pleurisy score of 4, exhibiting a bacterial load of 8.16 × 10^2^ ± 1.62 × 10^3^, slightly higher than *P. multocida* and *A. pleuropneumoniae* in this group (Figure 1).

### 3.6. Levels of IgG Anti-M. hyopneumoniae During Finishing Phase

**Figure 2:**
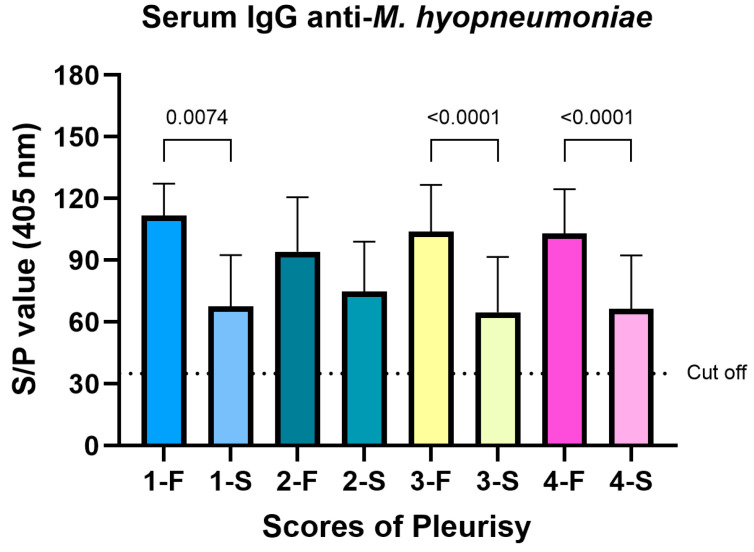
Comparison of serum IgG levels against *M. hyopneumoniae* across pleurisy scores (SPES) between samples collected at the farm (F) and slaughterhouse (S). A cut-off of 35 was set following the manufacturer’s guidelines.

The serology profile against *M. hyopneumoniae* was evaluated. For each pleurisy score (SPES 1 to 4), samples taken at the farm consistently showed higher IgG levels (S/P values) than those taken at the slaughterhouse. There was a significant difference between farm and slaughterhouse samples, with farm samples showing higher IgG levels (*p* = 0.0074) in pigs with a pleurisy score of 1, and the differences were highly significant, with *p*-values < 0.0001, indicating much higher IgG levels in farm samples compared to slaughterhouse samples in pigs classified with the most severe pleurisy scores of 3 and 4 (Figure 2).

### 3.7. Correlation and Mixed-Model Analysis

Correlations were found between the quantification of *M. hyopneumoniae* in lungs and the pleurisy score, with a significant positive correlation (*p* < 0.0001, r = 0.3284), indicating that higher pleurisy scores were associated with increased *M. hyopneumoniae* loads in lung samples, which also exhibited a positive correlation (*p* = 0.0002, r = 0.2741).

**Figure 3:**
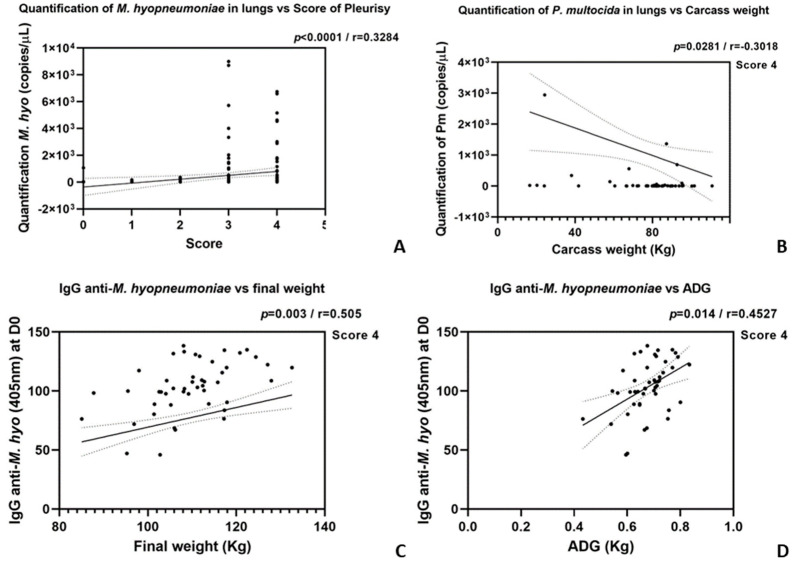
Correlations between lung health markers, pathogen presence, carcass weight, and immune response indicators in pigs, with statistical significance (*p*-values) and correlation coefficients (r) displayed for each graph. The solid line represents the linear regression, and the dashed lines indicate the confidence interval (CI 95%).

There was a weak negative correlation (*p* = 0.0376, r = −0.2491) between lung abscess presence and carcass weight, suggesting that pigs with abscesses tended to have lower carcass weights, particularly those with a pleurisy score of 3, which also exhibited a significant negative correlation (*p* = 0.0281, r = −0.3018). Also, there was a positive correlation (*p* = 0.014, r = 0.4527) between serum IgG levels against *M. hyopneumoniae* and the ADG, suggesting that pigs with higher IgG levels also had higher growth rates in samples with a pleurisy score of 4. A significant positive correlation (*p* = 0.003, r = 0.505) was observed between IgG levels and final weight, indicating that a higher immune response to *M. hyopneumoniae* correlated with greater final weights at pleurisy score 4. The graphs are presented in Figure 3. Additionally, a positive correlation was found between the quantification of App in lung lesions and females (*p* = 0.04, r = 0.452).

The model converged successfully (REML criterion = −226.6). The random effect variance for pen was estimated as zero (*σ*^2^_*Pen*_ = 0.00), indicating no significant contribution of differences between pens to the variance in the ADG. The residual variance was estimated at 0.006 (*σ*^2^_*Residual*_ = 0.006).

The fixed effect for gender suggests that males had a slightly higher ADG than females, with an estimated increase of 0.017 kg/day, but this effect was not statistically significant (t = 1.39, *p* > 0.05). The fixed effect for score suggests a negligible negative association between the lesion score and ADG (β = −0.0014), which was also not statistically significant (t = −0.19, *p* > 0.05) (Table 5).

An analysis of variance (ANOVA) was performed to assess the overall significance of the fixed effects in the model (Table 6). These results indicate that neither the fixed effect of gender nor score significantly contributed to explaining the variance in the ADG.

### 3.8. Profit and Economic Analysis

Regarding the economic analysis and the disease impact on profitability, in our model, the slaughter weight was affected by these factors: as the pleurisy score increased, the average slaughter weight slightly decreased, from 111.243 kg (score 2) to 109.734 kg (score 4), and the cost per kilogram increased with higher pleurisy scores, from USD 1.29 (score 2) to USD 1.32 (score 4), indicating potentially higher production costs associated with more severe health issues.

Additionally, the total revenue also declined with higher pleurisy scores, from USD 763.129 (score 2) to USD 752.775 (score 4), with a difference of USD 10.36 suggesting a financial impact due to reduced productivity and higher health management costs. The economic profit decreased significantly as pleurisy scores increased, from USD 7.79 for score 2 to USD 5.70 for score 4, indicating that pigs with higher pleurisy scores were less profitable. The benefit–cost ratio (BCR) remained constant at 0.21 across all scores, suggesting that while profit decreased, the ratio of benefits to costs was consistent, likely due to proportionate changes in revenue and costs. Finally, the return on investment (ROI) dropped from 5.33% (score 2) to 3.90% (score 4), showing reduced investment efficiency with higher pleurisy scores (Table 7).

## 4. Discussion

Pneumonias and pleurisies, caused by respiratory diseases, have a significant financial impact on pig production due to their direct effects on carcass quality and aggregate value. The severity of lung lesions in pigs often results in reduced weight gain during the finishing period, leading to a lower final weight at slaughter [35]. Since carcass pricing is directly influenced by weight, this loss in weight translates to a reduced financial return per animal. Moreover, carcasses with severe lesions are often diverted for additional inspections or, in some cases, condemned or directed to lower-value markets, which reduces the economic viability of slaughter. At some processing facilities, carcasses with severe pleurisy or substantial lung damage may be downgraded or sold at reduced prices, directly impacting total revenue. Therefore, the presence and severity of respiratory lesions affect not only efficiency and growth but also operational costs, as they demand greater investment in management and treatment. Consequently, minimizing respiratory disease in pigs is essential to improving profitability by optimizing carcass quality and maximizing total revenue [36].

The lesions observed in the lungs and carcasses at slaughterhouses can provide meaningful insights regarding the health conditions of the pigs since the beginning of the growing and finishing phase at the farm. The findings reveal a complex correlation between the pathogens *A. pleuropneumoniae*, *M. hyopneumoniae*, and *P. multocida* and the severity of pleuritic and pneumonic lesions observed in slaughtered pigs. Pleuritic lesions and pneumonic lesions are mainly found in the cranioventral areas of the lung when associated with *M. hyopneumoniae* [37]. The findings highlight that co-infections with these pathogens create a compounded effect on lesion severity, leading to an escalation in pleurisy scores and an increase in cranioventral pulmonary consolidations (CVPCs) and abscesses. In our study, the most severe pleurisy scores (SPES scores 3 and 4) were present in 28.7% and 13.4% of the carcasses, respectively, which underscores the high incidence of severe pleuritic lesions due to pathogenic synergy.

A strong correlation between *A. pleuropneumoniae* and *M. hyopneumoniae* was observed in studies analyzing pleurisy and pulmonary lesions. Petri et al. (2023) [6] examined 106 carcasses with varying pleurisy scores, finding herd-level pleurisy rates of 5.95–18.92% and pulmonary consolidation ranging from 5.21% to 16.32%. The average PPI values increased from score 2 (3.15–3.51) to score 4 (11.99–14.15), indicating an association with bronchopneumonia. Severe dorsocaudal pleurisy, particularly presenting as scores 3 and 4, frequently coincided with pulmonary lesions, reinforcing the role of co-infection by *M. hyopneumoniae*, *A. pleuropneumoniae*, and *P. multocida*, as we found in our study.

Merialdi et al. (2012) [4] reported dorsocaudal pleuropneumonia in 25.1% of lungs, with consistent SPES values (95% CI 0.78–0.86), demonstrating uniform pleurisy scoring at slaughter. The *A. pleuropneumoniae* pleuropneumonia index varied from 0 to 1.84, providing insights into prevalence. Also, qPCR analyses detected *A. pleuropneumoniae* in pleurisy lesions but lacked serological profiling, further associating the pathogen with pleuritis and increased carcass condemnation.

Petri et al. (2023) [6] quantified *M. hyopneumoniae* and *A. pleuropneumoniae* in pleurisy scores 0 to 4, highlighting their association with *P. multocida*. Similarly, Arruda et al. (2024) [10] showed that rising pleurisy scores correlated with increased *A. pleuropneumoniae* DNA loads and macroscopic and microscopic lung lesions. These findings reinforce the link between the pathogen load and lesion severity, directly contributing to carcass condemnations. Severe pleuritic and pneumonic lesions often require additional processing or redirection to lower-value markets, reducing economic returns for producers.

Our findings by analyzing data from diseased pigs reveal that severe lesions, particularly higher pleurisy scores, increase production costs. These costs stem from reduced average daily gain (ADG), lower carcass weights, and the diversion or condemnation of damaged carcasses, all of which directly impact revenue. As pleurisy scores increased, the cost per kilogram increased from USD 1.29 to USD 1.32 (scores 2 to 4), reflecting higher health management costs and decreased feed efficiency. Total revenue declined from USD 763,129 to USD 752,775, while the ROI dropped from 5.33% to 3.90%, demonstrating the dual impact of higher operational costs and reduced investment efficiency.

Zotti et al. (2012) [38] reported similar economic consequences of pleuritis, finding a 4.41 kg reduction in slaughter weight and 2.45 kg less lean meat in affected pigs compared to healthy ones. Affected pigs weighed 80.28 kg versus 84.64 kg for healthy pigs, with lean meat percentages of 56.61% and 58.06%, respectively, emphasizing profitability losses in the protein production chain. Further studies link lesions in specific lung areas to carcass weight reductions of 2.77 kg (right apical lobe) and 2.29 kg (dorsocaudal pleurisy). Financial impacts ranged from EUR 2.62 per pig for mild lesions to EUR 11.53 for severe lesions, underlining the substantial economic burden on pork production [8].

Histopathological analysis revealed that acute mixed bronchopneumonia was the most prevalent lung lesion (44.9%), while bronchus-associated lymphoid tissue (BALT) hyperplasia was observed in 98.5% of lungs. These findings align with previous studies reporting high rates of pulmonary lesions in Brazilian slaughtered pigs. One study analyzed 315 lungs with pulmonary consolidation, identifying suppurative bronchopneumonia in 48.9%, broncho-interstitial bronchopneumonia in 31.1%, and BALT hyperplasia in 81.6% [37]. Another study reported BALT hyperplasia in 98.2% and acute mixed bronchopneumonia in 55.3% of lungs from pigs with pleurisy [10]. These lesions indicate both acute infections and chronic inflammatory responses, highlighting challenges in controlling respiratory diseases under production conditions. Such lesions contribute to reduced final weight and increased production costs due to greater investment in medications and health management to mitigate their impact. Wallgren et al. [39] reported that, although the prevalence of CVPC and pleurisy at slaughter can be quite similar between farms, the pathogens, as well as the infection patterns during the fattening period, can be quite variable.

Respiratory diseases in fattening pigs reduce the ADG and increase the prevalence of severe lesions, meaning that these animals fail to reach optimal slaughter weight within the same timeframe as healthier pigs, increasing production costs and reducing feed efficiency. Pigs with higher pleurisy scores, especially those with a score of 4, showed a significantly lower ADG compared to animals with milder or no lesions. This effect is intensified in animals with more severe lesions, which require more time and resources to reach adequate weight, leading to a decline in return on investment (ROI) and overall profitability of the operation. Thus, effective pleurisy management, especially in male pigs, is essential for improving productive performance, maximizing the ADG, and reducing the financial impact associated with severe pulmonary lesions.

Moreover, a positive correlation was observed between IgG levels specific to *M. hyopneumoniae* and pig growth, measured by the ADG. This finding may suggest that an effective immune response against *M. hyopneumoniae* may help maintain growth performance despite respiratory infections. Vaccinated pigs with higher IgG levels showed resilience, limiting the severity of lung lesions and improving health outcomes. Conversely, non-vaccinated pigs in *M. hyopneumoniae*-seropositive farms exhibited higher lung lesion severity, as shown by Garcia-Morante et al. (2017) [40]. This suggests that enhancing pigs’ immune responses, through strategies such as vaccination and improved environmental conditions, could be an effective approach to reducing the impact of respiratory diseases and promoting healthy growth.

Regarding the analysis of mixed models, the estimated variance for the random effect of pen was zero, suggesting that there were no meaningful differences between pens in their contribution to the ADG in our study. This is likely due to homogeneous management conditions across the eight pens of our epidemiological unit. Regarding the fixed effects, male pigs had a slightly higher estimated ADG than females; however, this difference was not statistically significant. In addition, pleurisy scores were not significantly associated with the ADG. This suggests that the impact of lesions, as measured in this study, is negligible for the observed variability in the ADG. The lack of significance may be attributed to variability within groups or the relatively small effect size. This may indicate that other factors, such as genetic variability, nutrition, or environmental conditions, could play a more critical role in determining the ADG.

Although our results bring relevant and important information regarding respiratory pathogen impact and pneumonia and pleurisy lesions, some downfalls must be considered. First, sadly, we could not sample animals classified with a pleurisy score of 0 and pneumonia at the slaughterhouse, which limits the comparison of the results to animals in good health. Additionally, a limited number of carcasses scored 1, which made the health–economic analysis difficult. Secondly, we cannot discard the involvement of other respiratory pathogens, such as *G. parasuis* and *S. suis*, in the disease context and its association with the occurrence of lung lesions and pleuritis severity. In addition, respiratory infections caused by viruses such as swine influenza virus (IAV) and porcine circovirus type 2 (PCV-2) were not assessed in this study and should be considered for future studies to have a more comprehensive understanding of the subject and its associations with economic impact. Additionally, our study highlights routine antimicrobial use in swine production for disease prevention, a practice increasingly scrutinized for driving antimicrobial resistance. Future research should assess the economic impact of alternative strategies to promote sustainable practices that balance reduced antimicrobial use with animal health and productivity.

In summary, each pathogen plays a critical role in the progression and aggravation of respiratory lesions, with *A. pleuropneumoniae* leading to severe pleuritic lesions, *M. hyopneumoniae* contributing to chronic consolidation, and *P. multocida* amplifying overall lesion severity through abscess formation. The presence of these pathogens, especially in combination, significantly impacts both animal health and economic viability by increasing carcass condemnation rates and decreasing the quality of pork available for sale. Effective control and prevention strategies targeting these pathogens are essential to minimize respiratory disease severity and maximize carcass value at slaughter.

## 5. Conclusions

Our study highlights the significant health and economic impacts of respiratory diseases, particularly pleurisy and pneumonia, on finishing pigs. The association between pleurisy severity and reduced average daily gain, coupled with financial losses due to increased carcass condemnations, underscores the need for targeted interventions in swine health management. For the swine industry, implementing integrated strategies that include regular health monitoring; early disease detection through diagnostic tools like qPCR; and preventive vaccination programs, particularly for pathogens like *M. hyopneumoniae* and *A. pleuropneumoniae*, could substantially reduce disease prevalence and severity. Additionally, improving biosecurity measures, optimizing environmental conditions, and promoting the welfare of pigs during their growth stages may prevent disease spread and lower the incidence of co-infections, which aggravate respiratory conditions.

From a financial perspective, using these strategies can help producers reduce costs and improve production, which leads to higher profits. Overall, the findings underscore the importance of proactive health management as an integral component of sustainable pig farming, aligning advancements in animal health with economic outcomes.

## Figures and Tables

**Table 1:** Summary of the study sample size results.

Description	Number of Pigs
Lungs macroscopically evaluated	867
Pigs weighed at farm and at slaughter	163
Samples collected at DIF *	205
Data of animals used for economic analysis	156

* DIF: Final Inspection Department.

**Table 2:** Evaluation of body weight at the beginning of the study on a pig farm and at the end of the study at the slaughterhouse, the average daily gain (ADG) during the period, and the carcass weight after slaughter according to respective days of evaluation and scores of SPES.

Score of Pleurisy (SPES)	No Sampled Pigs (n)	Body Weight at Housing (Kg)	Body Weight at Slaughter (Kg)	Average Daily Gain (ADG)	Carcass Weight (Kg)	Meatiness (%)
1	7	32.11 (±6.37) ^a^*	114.5 (±10.36) ^a^	0.675 (±0.07) ^a^	59.51 (±6.8) ^a^	66.80 (±6.03) ^a^
2	30	28.69 (±3.7) ^ab^	111.5 (±8.16) ^a^	0.679 (±0.07) ^a^	69.78 (±25.69) ^a^	61.48 (±23.03) ^a^
3	70	27.55 (±3.86) ^b^	110.6 (±10.8) ^a^	0.681 (±0.08) ^a^	77.29 (±16.64) ^a^	70.79 (±14.17) ^a^
4	56	26.94 (± 4.04) ^b^	109.7 (±10.2) ^a^	0.678 (±0.08) ^a^	76.4 (±20) ^a^	69.37 (±16.39) ^a^
	N = 163	*p* = 0.007	*p* = 0.634	*p* = 0.998	*p* = 0.07	*p* = 0.13

* Means followed by different letters (a, b) in the column differ significantly by the Kruskal–Wallis test (*p* < 0.05).

**Table 3:** Histopathological findings in the 205 lung samples from the pigs evaluated in the study.

Microscopical Lung Lesion	Percentage (%)
Necrotizing pneumonia (NP)	0.97 (2/205)
Acute suppurative bronchopneumonia (ASBP)	5.37 (11/205)
Acute mixed bronchopneumonia (AMBP)	44.9 (92/205)
Non-suppurative acute bronchopneumonia (NSABP)	33.7 (69/205)
Subacute bronchopneumonia (SUBBP)	2.44 (5/205)
Chronic bronchopneumonia (CRBP)	8.29 (17/205)
Epithelial hyperplasia (EPH)	8.29 (17/205)
BALT	
0	1.46 (3/205)
+	30.5 (62/203)
++	45.8 (93/203)
+++	20.2 (41/203)
++++	2.9 (6/203)
Total scored between + to ++++	98.5 (202/205)

BALT: bronchial-associated lymphoid tissue hyperplasia; BALT hyperplasia was classified as absent (0), mild (+), moderate (++), marked (+++), or extensive (++++).

**Table 4:** Bacterial loads (gene copies/µL) of *Mycoplasma* (*M.*) *hyopneumoniae*, *Actinobacillus* (*A.*) *pleuropneumoniae*, and *Pasteurella* (*P.*) *multocida* in lung and pleural tissues were measured across different pleurisy scores (SPES) and the mean (± standard deviation) values are presented. Significant differences (*p* < 0.05) are noted with distinct letters in the same column.

Score of Pleurisy (SPES)	*M. hyopneumoniae* (±sd)		*A. pleuropneumoniae* (±sd)		*P. multocida* (±sd)	
	Lung	Pleura	Lung	Pleura	Lung	Pleura
1	4.27 × 10^1^ (±5.19 × 10^1^) ^ab^*	1.79 × 10^1^ (±1.32 × 10^1^)	5.37 × 10^1^ (±5.22 × 10^1^)	1.30 × 10^2^ (±2.64 × 10^2^)	2.78 × 10^1^ (±5.48 × 10^1^)	1.46 × 10^1^ (±1.05 × 10^1^)
2	3.86 × 10^1^ (±8.30 × 10^1^) ^b^	2.66 × 10^2^ (±7.64 × 10^2^)	8.07 × 10^1^ (±1.60 × 10^2^)	9.24 × 10^1^ (±1.79 × 10^2^)	3.83 × 10^1^ (±1.24 × 10^2^)	2.32 × 10^2^ (±1.29 × 10^3^)
3	7.0 × 10^2^ (±1.94 × 10^3^) ^a^	6.58 × 10^1^ (±2.71 × 10^2^)	3.23 × 10^3^ (±2.84 × 10^4^)	2.90 × 10^3^ (±1.79 × 10^4^)	2.30 × 10^4^ (±1.76 × 10^5^)	1.23 × 10^2^ (±6.02 × 10^2^)
4	8.16 × 10^2^ (±1.62 × 10^3^) ^a^	7.90 × 10^1^ (±3.03 × 10^2^)	8.39 × 10^1^ (±1.44 × 10^2^)	9.86 × 10^1^ (±2.12 × 10^2^)	1.13 × 10^2^ (±4.38 × 10^2^)	1.86 × 10^2^ (±8.20 × 10^2^)

* Means followed by different letters in the column differ significantly by the Kruskal–Wallis test (*p* < 0.05). The different letters (a, b) indicate significance.

**Table 5:** Fixed effects of the linear mixed-effect model evaluating factors influencing average daily gain (ADG) in pigs. The model includes gender and score as fixed effects and pen as a random effect. The intercept represents the baseline ADG for female pigs with a pleurisy score of 1. Estimates, standard errors, and *t*-values are shown.

Parameter	Estimate (*β*)	Standard Error	*t*-Value	Significance (*p*)
Intercept	0.6747	0.0237	28.48	<0.001
Gender (male)	0.0174	0.0125	1.39	0.790
Score of pleurisy	−0.0014	0.0075	−0.19	0.844

**Table 6:** Analysis of variance (ANOVA) for fixed effects in the linear mixed-effect model evaluating average daily gain (ADG). The table shows the sum of squares, mean squares, F-values, and significance levels for gender and score. Results indicate that neither factor significantly explained variability in ADG.

Effect	Sum of Squares	Mean Square	F-Value	Significance (*p*)
Gender	0.0118	0.0118	1.904	0.803
Score of pleurisy	0.0002	0.0002	0.037	0.845

**Table 7:** Economic data for pigs with pleurisy scores of 2, 3, and 4, focusing on slaughter weight, cost per kilogram, total revenue, economic profit, benefit–cost ratio (BCR), and return on investment (ROI).

Score of Pleurisy (N = 156)	Slaughter Weight (kg)	Cost Per Kg (USD)	Total Revenue	Economic Profit (USD)	BCR (USD)	ROI (%)
2 (n = 30)	111.243	1.29	763.129	7.79	0.210	5.33
3 (n = 70)	110.616	1.32	758.824	6.92	0.214	4.73
4 (n = 56)	109.734	1.32	752.775	5.70	0.207	3.90

## Data Availability

Data are contained within the article and Supplementary Materials.

## Supplementary Materials

The following supporting information can be downloaded at: www.mdpi.com/article/10.3390/vetsci11120668/s1, Table S1: Slaughterhouse Pleurisy Evaluation System (SPES) [23]; Table S2: Score and extent of consolidation lesion in each pulmonary lobe [24]; Figure S1: Bar plots of *Mycoplasma hyopneumoniae*, *Actinobacillus pleuropneumoniae*, and *Pasteurella multocida* detection with estimated quantification per µL by qPCR. (A), (B) and (C) estimated quantification of *M. hyopneumoniae*, *A. pleuropneumoniae*, and *P. multocida* in lung tissues, respectively; (D), (E), and (F) estimated quantification in parietal pleura samples. Biological samples were classified according to the pleurisy scores (1 to 4). Kruskal–Wallis test was used (*p* < 0.05).
