# Supplementary material for: Health–Economic Impact Attributable to Occurrence of Pleurisy and Pneumonia Lesions in Finishing Pigs
Source: Vet Sci. 2024 Dec 20;11(12):668. doi: 10.3390/vetsci11120668 (PMC11680166; doi:10.3390/vetsci11120668)
Supplement: Supplementary file 1 [file vetsci-11-00668-s001.zip › vetsci-3334683-supplementary.pdf]

Table S1: Slaughterhouse Pleurisy Evaluation System (SPES) [23].

Score	Pleuritis and SPES
0	Absence of macroscopic lesions
1	Adhesion between the cranioventral portions of the apical, cardiac, and diaphragmatic lobes, or amild unilateral adhesion on the ventral margin of the diaphragmatic lobe
2	Adhesions with small to moderate extension on one of the diaphragmatic lobes
3	Like score 2, but bilateral, and in one of the diaphragmatic lobes, it may present extensive lesions
4	Severe extension lesion, affecting at least 1/3 of both diaphragmatic lobes

Table S2: Score and extent of consolidation lesion in each pulmonary lobe [24].

Scores	Extension of lung lesion by lobe (% of lung area)
0	Normal lobe, no consolidated lesion
1	<25% of the affected lobe surface
2	25 a 50% of the affected lobe surface
3	51 a 75% of the affected lobe surface
4	>76% of the affected lobe surface

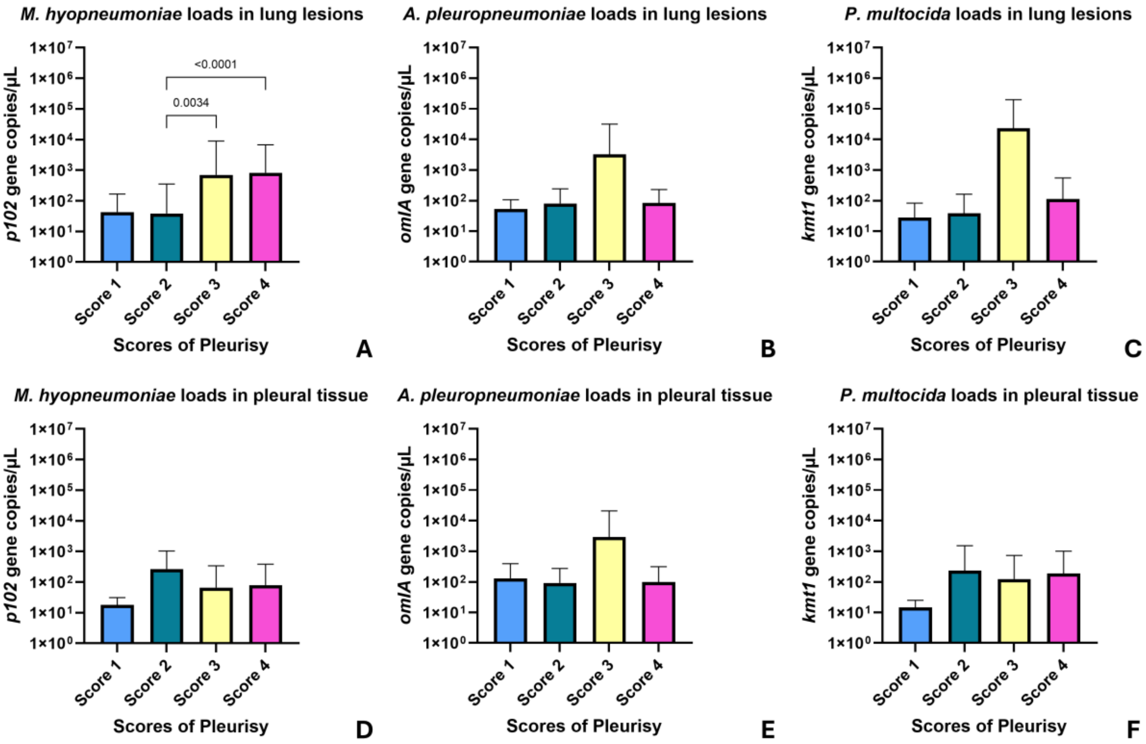

Figure S1: Bar plots of *Mycoplasma hyopneumoniae*, *Actinobacillus pleuropneumoniae* and *Pasteurella multocida* detection with estimated quantification per µL by qPCR. (A), (B) and (C) estimated quantification of *M. hyopneumoniae*, *A. pleuropneumoniae* and *P. multocida* in lung tissues, respectively; (D), (E) and (F) estimated quantification in parietal pleura samples. Biological samples were classified according to the pleurisy scores (1 to 4). Kruskall-Wallis test were used ($p<0.05$).
